# Anti-Fibrotic Effects of RF Electric Currents

**DOI:** 10.3390/ijms241310986

**Published:** 2023-07-01

**Authors:** María Luisa Hernández-Bule, Elena Toledano-Macías, Luis Alfonso Pérez-González, María Antonia Martínez-Pascual, Montserrat Fernández-Guarino

**Affiliations:** 1 Bioelectromagnetic Laboratory, Instituto Ramón y Cajal de Investigación Sanitaria (Irycis), Carretera de Colmenar Viejo, km. 9.100, 28034 Madrid, Spain; elena.toledano@hrc.es (E.T.-M.); m.antonia.martinez@hrc.es (M.A.M.-P.); 2 Dermatology Service, Hospital Ramón y Cajal, Instituto Ramón y Cajal de Investigación Sanitaria (Irycis), Carretera de Colmenar Viejo, km. 9.100, 28034 Madrid, Spain; pg.l.alfonso@gmail.com (L.A.P.-G.); drafernandezguarino@gmail.com (M.F.-G.)

**Keywords:** fibrosis, hypertrophic scar, keloids, radiofrequency, myofibroblast, metalloproteinase, extracellular matrix proteins, MAP-Kinases, NFκB

## Abstract

Hypertrophic scars and keloids are two different manifestations of excessive dermal fibrosis and are caused by an alteration in the normal wound-healing process. Treatment with radiofrequency (RF)-based therapies has proven to be useful in reducing hypertrophic scars. In this study, the effect of one of these radiofrequency therapies, Capacitive Resistive Electrical Transfer Therapy (CRET) on biomarkers of skin fibrosis was investigated. For this, in cultures of human myofibroblasts treated with CRET therapy or sham-treated, proliferation (XTT Assay), apoptosis (TUNEL Assay), and cell migration (Wound Closure Assay) were analyzed. Furthermore, in these cultures the expression and/or localization of extracellular matrix proteins such as α-SMA, Col I, Col III (immunofluorescence), metalloproteinases MMP1 and MMP9, MAP kinase ERK1/2, and the transcription factor NFκB were also investigated (immunoblot). The results have revealed that CRET decreases the expression of extracellular matrix proteins, modifies the expression of the metalloproteinase MMP9, and reduces the activation of NFκB with respect to controls, suggesting that this therapy could be useful for the treatment of fibrotic pathologies.

## 1. Introduction

The wound-healing process comprises a complex succession of events that is organized into three consecutive phases: inflammation phase, proliferation phase, and remodeling phase [1,2,3].

During the proliferation phase, fibroblasts are activated and acquire the capacity to express alpha smooth muscle actin (α-SMA), differentiating into myofibroblasts. These differentiated fibroblasts allow the wound to contract and close. Once the wound is closed, under normal physiological conditions, the myofibroblasts enter into apoptosis and are replaced by fibroblasts. In wound healing, various biochemical/cellular signals, such as Wnt and Notch signaling, AKT/mTOR, transforming growth factor beta (TGF-β), mitogen-activated protein kinase (MAPK), or binding protein to the nuclear factor kappa enhancer (NF-ĸB) intervene in a tightly coordinated cascade to repair the damage [4]. When this process is deregulated, abnormal or excessive scarring can occur, leading to the development of cutaneous fibrosis, which clinically manifests as hypertrophic and keloid scars.

Both types of fibrosis are differentiated by their etiology, symptoms, causes, and location, among others [3]. The main causes of hypertrophic scars are surgery, severe burns, trauma, or even insect bites, while keloids usually develop several months or even years after an injury or inflammation of the skin, and can be due to minor skin damage such as piercings, vaccinations, or acne [5]. Although the etiology of keloids is unknown, a strongly associated genetic, racial, and anatomical component has been observed, since they are more frequent in African Americans and Asians, and in the upper part of the thorax or earlobe [6]. Chronic infections and repeated injuries can also promote hypertrophic scar formation and tissue fibrosis [7].

At the tissue level, hypertrophic scars and keloids present hyperplasia, swelling, and redness, with aberrant growth being a specific characteristic of keloids, which usually invades beyond the margins of the original wound, unlike hypertrophic scars, which remain circumscribed to the original margins of the lesion [8]. In both pathologies, the myofibroblasts are not replaced by fibroblasts, but remain in the proliferating lesion with low rates of apoptosis [6]. These myofibroblasts generate persistent chronic inflammation, due to the continuous production of profibrotic cytokines and chemokines, such as TGF-β1, TGF-β2, VEGF, FGF, and CTGF [9], and synthesize a large amount of fibrotic extracellular matrix, composed by collagen types I and III, and α-SMA, which prevents the normal functioning of the affected tissues [10]. This dysfunction causes itching and pain in most patients for long periods of time [11]. In addition, from a psychological point of view, these pathologies generate a negative cosmetic and emotional impact on patients, which can even limit their social interaction and affect their self-esteem [12].

Currently, hypertrophic scars and keloids represent a great challenge for dermatologists and surgeons due to the lack of effective specific treatments. Corticosteroid administration, surgical excision, silicone gels, local radiotherapy, hormonal therapy, or laser have been the conventional treatments, but often do not achieve complete cure, especially in the case of keloids due to high rates of recurrence [13]. There is experimental evidence that hyperthermia induced by radiofrequency (RF) is effective in scar or keloid regeneration treatments. In this regard, clinical evidence indicates that the application of RF [14,15], alone or in combination with other physical therapies such as laser [16] or ultrasound [17], can be effective for the treatment of hypertrophic and keloid scars. Thus, physical therapies induce a remodeling of the collagen derived from the denaturation of the fibers due to hyperthermia. In particular, therapies using capacitive RF have been shown to be useful for reducing hypertrophic scars and increasing the flexibility of fibrotic skin tissue [18]. One of these capacitive therapies is Capacitive Resistive Electrical Transfer Therapy (CRET). This non-invasive therapy uses frequency currents around 0.5 MHz to induce deep heating in the treated tissues. Under thermal conditions, it has been shown to be effective in skin regeneration in animal models through increased skin thickening and an increase in collagen production and angiogenesis [19]. Additionally, previous research by our group has revealed that this therapy, under non-thermal conditions, can promote skin regeneration through fibroblast migration and keratinocytes, fibroblasts, and stem cell proliferation [2,20], as well as induce differentiation [21]. These effects would be mediated by changes in the expression and localization of intercellular adhesion proteins or cell–substrate adhesion proteins, and in kinases involved in cell proliferation and migration. In this way, electrical stimulation would promote the formation of granulation tissue before the closure of the external tissue layers, thus avoiding abnormal healing of the wound or its chronification.

However, in relation to fibrotic processes, besides cases of favorable effects of CRET observed in the clinic, the published experimental evidence is null and the biological bases of these effects are unknown. Given the absence of effective strategies for the treatment of these fibroses, it is of great interest to promote new therapeutic alternatives to the existing ones for their treatment. On this basis, the objective of this study was to investigate, in human cell models under normothermic conditions, the potential capacity of CRET to regenerate already formed hypertrophic or keloid scars and/or to prevent their formation in the initial phases of healing prior to their consolidation, in patients with a clinical predisposition to generate them.

## 2. Results

### 2.1. Extracellular Matrix Production

**Figure 1:**
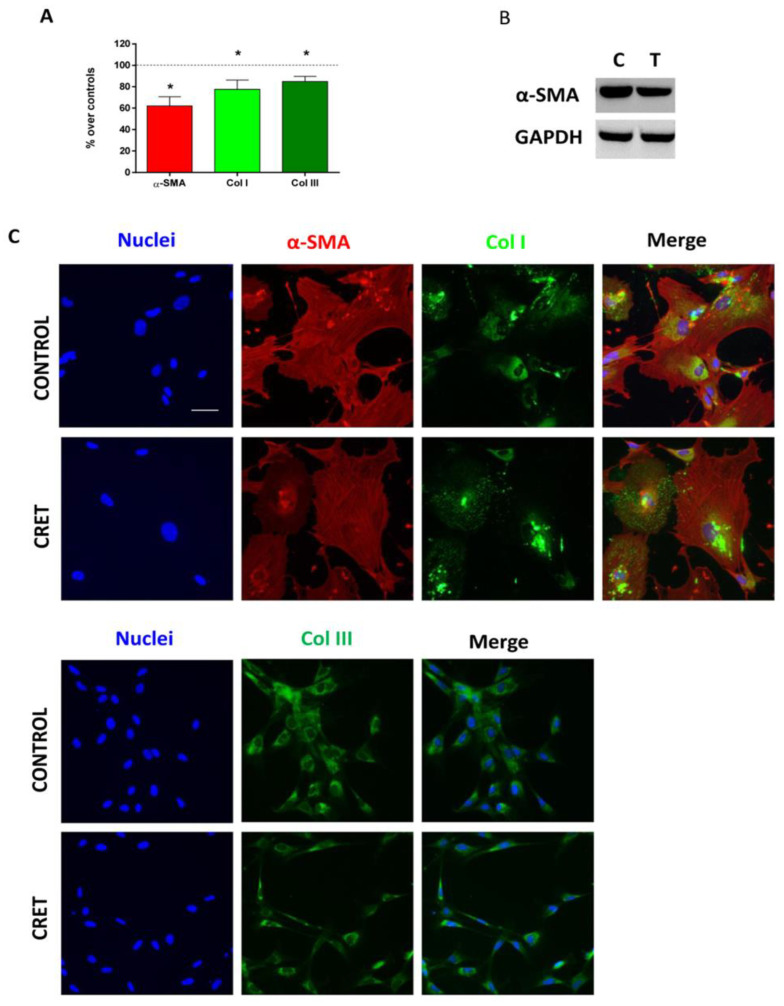
α-SMA, Col I and Col III expression. (**A**) α-SMA, Col I, and Col III protein expression of myofibroblasts at 48 h of CRET or sham treatment. Fluorescence intensity measurement per MHC channel. Data normalized over the corresponding sham-exposed controls (dashed line represents the control group: 100%). Means ± SEM of the fluorescence intensity/total nuclei of at least three experimental repeats per protein. *: 0.05 > *p* ≥ 0.01; Student’s *t*-test. (**B**) Inmunoblot for α-SMA. Representative blots at 48 h of CRET or sham treatment (100 µg protein/lane). GAPDH were used as loading control. C: Control. T: CRET treatment. (**C**) Immunofluorescence expression of α -SMA, Col I, Col III, and merged micrographs. Representative micrographs. Red: α-SMA, Green: Col I or Col III. Blue: cell nuclei. Scale bar: 20 µm.

The immunoblot revealed a statistically significant reduction of 20 ± 6.3% (*p* = 0.011; Student’s *t*-test) over the control in α-SMA expression, in cultures treated with CRET for 48 h (Figure 1A). This protein was also analyzed with immunofluorescence, and image analysis confirmed the decrease (38% over controls) in the amount of α-SMA in CRET-treated cultures versus controls (Figure 1B,C). Furthermore, collagen type I also decreased by 23% and collagen type III content by 16%, compared to the control. The results were statistically significant in all three analyses (Figure 1B,C).

### 2.2. Cell Proliferation and Death

**Figure 2:**
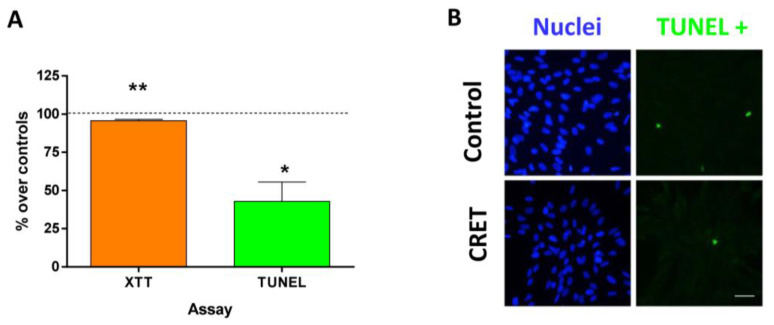
XTT and TUNEL assays. (**A**) Proliferative and apoptotic myofibroblast valuation after 48 h of intermittent CRET treatment. Data are means ± SEM normalized over the corresponding sham-exposed controls (dashed line represents the control group: 100%). Five experimental replicates of XTT assay and three experimental replicates of TUNEL assay. *: 0.01 ≤ *p* < 0.05; **: 0.001 ≤ *p* < 0.01. Student’s *t*-test. (**B**) Representative micrographs of TUNEL+ cells at 48 h from CRET or sham stimulation. Green: TUNEL+ cells; Blue: Nuclei stained with DAPI. Scale bar: 20 µm.

The myofibroblasts treated with CRET for 48 h decreased their proliferation compared to the control although very weakly (4% over control; statistically significant) (Figure 2A). On the other hand, electrically treated myofibroblasts significantly decreased the number of apoptotic cells, although the proportion of TUNEL+ cells was low in the cultures, and did not exceed 10% of the total cells counted in any of the replicates (Figure 2A,B).

### 2.3. Cell Migration

**Figure 3:**
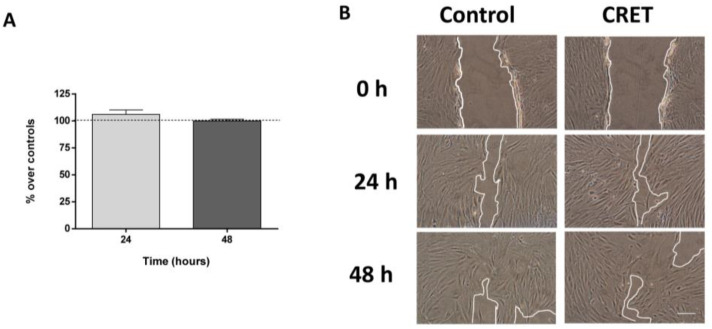
Wound Assay. (**A**) Computational quantification of the gap closure calculated as the mean distance (µm) between the edges in three equidistant points of the gap, using Photoshop software. Data are means ± SD, normalized over the corresponding controls (dashed line represents the control group: 100%), of five experimental replicates per time interval. NS: *p* ≥ 0.05; Student’s *t*-test. (**B**) Representative micrographs of the wound at *t* = 0, 24 and 48 h from CRET or sham treatment (control). Scale bar: 50 µm.

The results of the wound closure assay, performed at 24 and 48 h of CRET treatment, did not show statistically significant changes in the migration rate of electrically treated myofibroblasts compared to controls (Figure 3A,B).

### 2.4. Expression of Metalloproteinases MMP1 and MMP9

**Figure 4:**
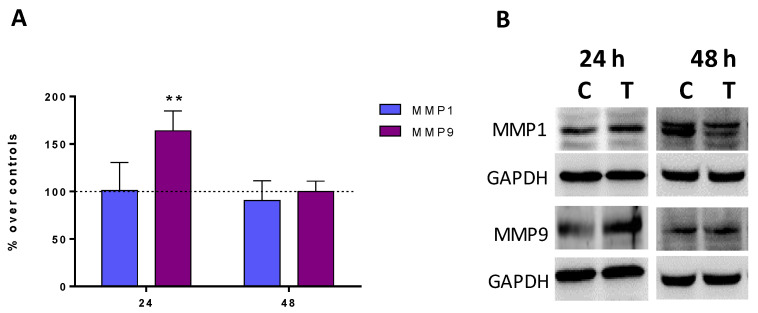
MMPs expression. (**A**) Densitometry values for MMP9 and MMP1 expressions at 24 or 48 h of CRET or sham treatment. Data normalized over the corresponding sham-treatment controls (dashed line represents the control group 100%). Means ± SD of the protein/GAPDH ratios of at least four experimental repeats per protein and time interval. **: 0.001 ≤ *p* < 0.01. Student’s *t*-test. (**B**) Representative blots at 24 h or 48 h of CRET or sham treatment (100 µg protein/lane). GAPDH were used as loading control. C: Control. T: CRET treatment.

At 24 h, the CRET treatment increased the expression of MMP9 by 63% compared to controls and the differences were statistically significant. On the contrary, 48 h treatments did not modify the expression of this protein compared to the control. Neither did the expression of MMP1 show changes with respect to the control, at any of the moments analyzed (Figure 4A,B).

### 2.5. Expression and Activation of MAP-Kinase ERK1/2 and Nuclear Factor (NF)-Kappa B p65

**Figure 5:**
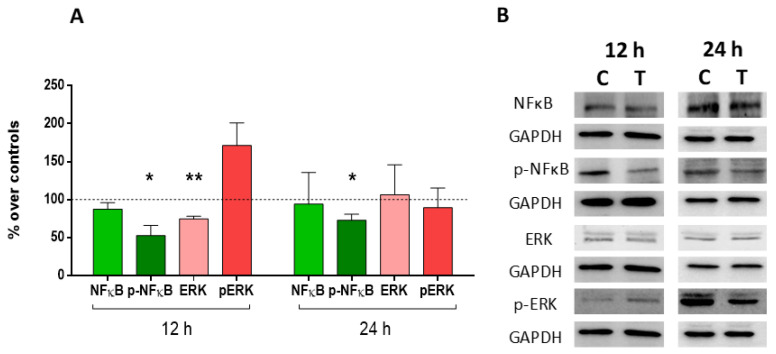
ERK1/2, p-ERK1/2, NFkB, and p-NFkB expression. (**A**) Densitometry values for ERK1/2, p-ERK1/2, NFkB, and p-NFkB expressions at 12 h or 24 h of CRET or sham treatment. Data normalized over the corresponding sham-exposed controls (dashed line represents the control group 100%). Means ± SD of the protein/GAPDH ratios of at least three experimental repeats per protein and time interval. **: 0.001 ≤ *p* < 0.01. *: 0.05 > *p* ≥ 0.01. Student’s *t*-test. (**B**) Representative blots at 12 h or 24 h of CRET or sham treatment (100 µg protein/lane). GAPDH were used as loading control. C: Control. T: CRET treatment.

The results of the immunoblot showed statistically significant decreases in the expression of ERK, compared to the control at 12 h, while at 24 h there were no changes in its expression. The active form of the protein (p-ERK) did not change its expression at 12 or 24 h compared to the control. On the other hand, the NFκB factor did not change its expression compared to controls, although its activation did. Thus, at both 12 and 24 h, the expression of p-NFκB decreased significantly with respect to the controls (Figure 5A,B).

## 3. Discussion

Cutaneous fibrosis is a skin pathology that involves pain, itching, lack of functionality and psychological problems for patients who suffer from it. The main treatments for hypertrophic scars or keloids are not considered fully satisfactory since they are not very effective, can cause pain, or have adverse side effects. Laser is one of the first line treatments, however, it is expensive and requires repeated sessions. Other alternatives, such as intralesional treatments with corticosteroids, bleomycin, or 5-fluorouracil are painful and require multiple infiltrations. Local radiotherapy is a good treatment alternative but it needs to be applied in specialized centers and in several sessions that produce the patient’s radiation. In any case, the approach needs to be multidirectional, which is why the combination of treatments is generally chosen. For this reason, these fibroses represent a challenge for medicine, and it is of great interest to find alternative therapies for their treatment.

Currently, progress is being made in understanding the molecular mechanism of these fibroses, which is generating new therapeutic strategies for the treatment of this pathology. Since the pathological characteristics of cutaneous fibrosis are characterized by an excess of extracellular matrix produced by fibroblasts at the tissue level, the action mechanisms of these new therapies aim to achieve one or more of the following objectives: reduction or inhibition of the proliferation of fibroblasts and myofibroblasts, reduction or inhibition of the production of extracellular matrix (collagen, α -SMA and fibronectin), increased apoptosis or necrosis of fibroblasts, decreased expression of TGF-β1 (a key regulator of collagen synthesis), decreased expression of the GFR β1/Smad2/3 signaling pathway, changes in the activity of metalloproteinases, decreased or modulated inflammation, and/or increased production of IFN-α (due to its antifibrogenic capacity or increased normal angiogenic activity) [22]. In this study we have investigated whether CRET therapy can act on one or several of these key action mechanisms in skin fibrosis.

Due to the relevance of extracellular matrix (ECM) production in the generation of the fibrotic process, we first investigated whether CRET could reduce the synthesis of matrix components or degrade the existing one. In fact, numerous investigations aimed at finding new pharmacological strategies focus on the reduction of collagen and extracellular matrix deposits such as hypertensives including the angiotensin-converting enzyme ACE-inhibitor [23], calcineurin inhibitors [24], doxorubicin [25], shock wave therapy [25,26], stem cell therapy, interferons [5], tamoxifen [27], or RNA-based therapies [28]. Some electrical therapies have also shown the ability to reduce collagen deposits in fibrotic processes through its degradation. Thus, electrical therapies with degenerate waves have shown an improvement in pain and itching, as well as a reduction in the formation of excess collagen in keloids [29]. In RF-based therapies, treatment of hypertrophic scars with moderate thermal increases (below 40 °C) generated by monopolar radiofrequency induces remodeling of collagen fibers, whether applied as a single therapy [14] or in association with negative pressure [18]. Other RF therapies have also shown changes in the morphology or production of collagen fiber [15] or a reduction in the amount of collagen I, collagen III, and TGF-β1 in scars, when RF is combined with pulsed light versus laser [16]. In our study conducted under subthermal conditions, CRET treatment of myofibroblasts significantly decreased the amount of collagen type I, type III, and α-SMA in their ECM. It is known that the changes induced in the connective tissue by RF-based therapies are not explained solely by the increase in temperature [30]. Given that the CRET therapy in our experiments has been applied under normothermia conditions (37 °C), the changes observed in the collagen content would not be related to denaturation and rearrangement of the fibers but to other molecular mechanisms such as the modulation of the activity of metalloproteinases (MMPs) or the Smad2/3 pathway.

Indeed, MMPs also seem to play a critical role in wound healing and scar formation, and their inhibition could be a strategy to treat fibrosis. MMPs are endopeptidases with a catalytic domain of Zn^2+^ ions that interact with multiple components of the extracellular matrix [31]. It has been reported that the expression of MMPs is elevated in skin lesions compared to intact skin, while hypertrophic scar fibroblasts appear to have low MMP-1 [32] and MMP9 [33] activity, which would favor the accumulation of collagen. For this reason, some novel therapies have sought to modulate the expression of MMPs. Thus, for example, shock waves have been shown to be capable of reducing the collagen fibers of keloids due to increases in the expression of MMP13 [25], while stem cell therapies attenuated the expression of tissue inhibitor of metalloproteinases 1 (TIMP-1) in keloid fibroblasts [34], which would promote the activity of MMPs. Similarly, pressure therapies were able to increase the expression of MMP9 and MMP12 in tissue from hypertrophic scars [35]. In this study, it was analyzed whether the observed decrease in electrically induced collagen expression and content could be due to a modulation in the expression of two of these MMPs: MMP1 and MMP9. The results showed that MMP9 increases significantly after 24 h of CRET treatment, while no changes in MMP1 expression were detected compared to the control. Thus, this activation of MMP9 could be responsible for the degradation of collagen I and collagen III observed after electrical stimulation, which would promote the reduction of fibrosis in CRET treatments.

Another of the objectives pursued by numerous emerging therapies for the treatment of fibrosis is the reduction of the proliferation of fibroblasts, responsible for most of the collagen and ECM deposition that occurs in wound healing, both normal and pathological. These actions are promoted by fibrogenic growth factors, such as TGF-β1, PDGF, fibroblast growth factor β (FGF-β), and insulin-like growth factor I (IGF-I). Fibroblasts isolated from keloid tissue have elevated expressions of TGF-β1, periostin, PAI-2, and inhibin beta A compared to normal healthy tissues [6]. For this reason, numerous therapies have focused on reducing the proliferation of fibroblasts such as the ACE-inhibitor lisinopril [23], botulinum toxin [36], calcineurin inhibitors [24], doxorubicin [37], microneedles [38], stem cell therapy [34,39], imidazoquinolines [40], or interferons [5]. CRET therapy has been shown to slightly reduce myofibroblast proliferation and decrease ERK1/2 expression relative to control, which could lead to an anti-proliferative effect. However, since no changes in the activation of the protein were observed, but only in its expression, and the effect on proliferation was weak, it is possible that such changes play a secondary role in the potential anti-fibrotic effect of the CRET therapy. Furthermore, in wound regeneration, proliferating mesenchymal stem cells (MSCs) play a key role, and recent studies have shown that exosomal microRNA derived from adipose-derived mesenchymal stem cells (ADSC) suppresses proliferation and exerts a pro-angiogenic role in keloid fibroblasts [41]. Therefore, a therapy that promotes stem cell proliferation could improve tissue fibrosis. This is the case of CRET therapy, which is capable of promoting the proliferation of ADSC [20]. Thus, CRET could also reduce or prevent the generation of hypertrophic scars by promoting stem cell proliferation while reducing that of myofibroblasts.

On the other hand, fibroblasts derived from hypertrophic scars overexpress transglutaminase that could inhibit their apoptosis, thus enhancing the fibrotic phenotype by increasing the number of cells capable of generating extracellular matrix [42]. For this reason, this enzyme has been the target of physical therapies such as degenerate electrical waveform stimulation in combination with photodynamic therapy [43]. In our study, treatment with CRET induced a decrease in apoptosis in cultures compared to those not treated electrically, although the actual proportion of apoptosis detected was very low. The proliferative phase of wound healing also involves the active migration of fibroblasts [6]. In our CRET assays, the myofibroblast migratory rate did not change with respect to control. Therefore, CRET would not significantly alter the migration and apoptosis processes of myofibroblasts.

The skin is considered a neuroendocrine organ that has immunological, endocrine, and neurological functions to maintain the body’s homeostasis. For this reason, its resident cells, and the circulating cells of the immune system express neuropeptides and neurotransmitters, and produce endocrine factors and cytokines in response to stress [44]. This organizational system has been called the cutaneous hypothalamus-pituitary-adrenal axis (cHPA) and its functions are regulated through two main signaling pathways: the corticotropin-releasing hormone (CRH) pathway, and the proopiomelancortin (POMC) pathway.

Skin cells activate the CRH signaling pathway under physiological, pathological conditions, or after stimulation with microbial antigens or UVB spectrum radiation. Through the CRH pathway, these cells regulate cell proliferation and differentiation, as well as activities of the immune or endocrine system [45]. CRH can act as a pro-inflammatory agent since it has been described that the activation of this CRH pathway stimulates the activity of NFkB [46], a key transcription factor that regulates a multitude of genes responsible for triggering the inflammatory response. NFkB has been shown to be activated in keratinocytes and fibroblasts from keloids [8]. Furthermore, in fibrosis, myofibroblasts generate persistent chronic inflammation, due to the continuous production of profibrotic cytokines and chemokines, such as TGF-β1, TGF-β2, VEGF, FGF, and CTGF [9].

NFkB is also modulated by the proopiomelanocortin POMC signaling pathway. In skin cells such as keratinocytes, NFkB is indirectly inhibited by α-melanocyte-stimulating hormones (α-MSH) [45], while in fibroblasts, α-MSH reduces NFkB activation [47]. On the other hand, POMC gene expression, and POMC peptides and proteins production, can be enhanced by electromagnetic radiation such as UVB, among others [45]. Recent studies show that therapies based on the POMC pathway could be a new therapeutic strategy for the treatment of diseases related to fibrotic conditions, since melanocortin drugs have been shown to be able to reduce collagen deposition, myofibroblast activation, and the production of pro-inflammatory mediators [47]. The results of our study show that CRET decreases the activation of NFkB in myofibroblasts, and reduces the expression of collagens through MMP9. Considering these results, and given that POMC and CRH are activated by electromagnetic radiation, it cannot be ruled out that CRET radiofrequency currents could also act through these POMC- or CRH-signaling pathways. If so, CRET physical treatment could reduce the pro-inflammatory response in myofibroblasts through this cutaneous neuroendocrine mechanism, without excluding other potential pathways yet to be studied.

## 4. Materials and Methods

### 4.1. Cell Culture

Human dermal fibroblasts (HF) isolated from neonatal foreskin (cat. no. C-004-5C, Thermo Fisher, Carlsbad, CA, USA) were seeded in medium composed of high-glucose D-MEM (Biowhittaker, Lonza, Verviers, Belgium) supplemented with 10% inactivated fetal bovine serum (Gibco, Waltham, MA, USA), 1% glutamine, and 1% penicillin-streptomycin (Gibco) and maintained in a 5% CO_2_ atmosphere at a temperature of 37 °C inside CO_2_ incubators (Thermo Fisher Scientific, Waltham, MA, USA). The cells were subcultured once a week.

HF were plated at 450 cells/cm^2^ density on the bottom of 60 mm Petri dishes (Nunc, Roskilde, Denmark), except for immunofluorescence assays, in which the cells were seeded on glass coverslips placed on the bottom of the plates. One day after seeding, cells were incubated with complete DMEM supplemented with 2 ng/mL of TGF-β1 (Peprotech, Rocky Hill, NJ, USA) for an additional 12 or 13 days depending on the experiment, renewing this conditioned culture medium every 4 days (Figure S1). Depending on the aim of the corresponding experiment, a total of 5 or 10 Petri dishes were used for experimental replicate.

### 4.2. Electric Treatment

The procedure for RF exposure has been described in detail elsewhere [48,49]. Briefly, 11 days after seeding, pairs of sterile stainless-steel electrodes designed ad hoc for in vitro stimulation were inserted in all Petri dishes and connected in series. Only the electrodes of dishes for electrical stimulation were energized using a signal generator (Indiba Activ HCR 902, INDIBA^®^, Barcelona, Spain), while the remaining plates were sham-exposed simultaneously inside an identical, separate CO_2_ incubator. The intermittent stimulation pattern consisted of 5-min pulses of 448 kHz, sine wave current delivered at subthermal densities of 100 µA/mm^2^, separated by 4-h interpulse lapses, and administered for a total of 12, 24, or 48 h. Such exposure parameters have been shown to stimulate the proliferation and early differentiation of human, adipose-derived stem cells [20,21,50] and in human dermal fibroblasts and keratinocytes [2].

### 4.3. XTT Proliferation Assay

Cell proliferation was determined with XTT assay (Roche, Switzerland). After 48 h of CRET or sham treatment, the cells were incubated for 3 h with the tetrazolium salt XTT in a 37 °C and 6.5% CO_2_ atmosphere, as recommended by the manufacturer. The cell culture confluence reached 60–70%. The metabolically active cells reduced XTT into colored formazan compounds that were quantified with a microplate reader (TECAN, Männedorf, Switzerland) at a 492 nm wavelength. At least 3 experimental replicates per cell type were conducted.

### 4.4. TUNEL Assay

Apoptosis in cultures was assessed with the TUNEL assay. For this, the DeadEnd^TM^ Fluorometric TUNEL System (Promega, Madison, WI, USA) was used. Cells treated for 48 h with CRET or sham exposure were fixed with 4% paraformaldehyde (Merck, Darmstadt, Germany) and then proceeded according to the manufacturer’s instructions. The fluorescence of apoptotic cells was detected with fluorescence microscopy. Nuclei were counterstained with ProLongTM Gold antifade reagent whit DAPI (Invitrogen, Eugene, OR, USA). TUNEL+ cells were assessed using an inverted fluorescence microscope (Nikon Eclipse Ts2R, Nikon, Tokyo, Japan) coupled to a digital camera (Nikon DS-Ri2). Photomicrographs were taken and the images were computer-analyzed with NIS-Elements Br image software (version 4.40, Nikon, Japan). TUNEL+ cell identification and quantification were based on fixed thresholds of fluorescence (MHC mode) determined and automated for all images at the beginning of the analysis. The values obtained were normalized with respect to the number of cells per field. Three experimental replicates were performed.

### 4.5. Wound Assay

In each experimental replicate of the wound closure assay, HF were seeded on 8 plates and incubated at 37 °C. After 10 days of incubation with TGF-β1, a wound was scratched on the confluent monolayer of each of the plates, using a glass pipette tip. Dishes were washed with PBS (Gibco) to eliminate debris, and the cultures were maintained in high-glucose D-MEM medium supplemented with 10% inactivated fetal bovine serum (Gibco), 1% glutamine, 1% penicillin-streptomycin (Gibco), and 2 ng/mL of TGF-β1. Next, 4 plates were exposed to CRET for 24 or 48 h, while the remaining plates were sham-exposed for the same intervals. At 0, 24 and 48 h after scratching, 6 micrographs were taken of 3 equidistant points in the wounds, using a digital camera (Nikon DS-Ri2) coupled to an inverted microscope (Nikon Eclipse Ts2R). The wound closure rate was determined with dual analyses of the obtained images, using standard Photoshop software (Adobe Photoshop CS3, Extended version 10.0). Five experimental replicates were performed.

### 4.6. Immunofluorescence for α-SMA, Collagen Type I and Collagen Type III

At the end of 48 h RF- or sham-exposure, the samples were fixed with 4% paraformaldehyde (Merck, Darmstadt, Germany) and incubated overnight at 4 °C with mouse monoclonal anti- α-smooth muscle actin antibody α-SMA (1:400; cat. no. A 2547; Sigma Aldrich, St. Louis, MO, USA), rabbit polyclonal anti-collagen type I antibody (1:400; cat. no. NB600-408-0.1 mg; Novus; Centennial, CO, USA) and rabbit polyclonal anti-collagen type III alpha 1 antibody (1:400, cat. no. NB600-594SS; Novus; CO, USA). Afterwards, the samples were fluorescence-stained with Alexa Fluor^TM^ 568 goat anti-mouse IgG (1:500; cat. no. A11031; Invitrogen; Eugene, OR, USA) or Alexa Fluor^TM^ 488 goat anti-rabbit IgG (1:500; cat. no. A11031; Invitrogen) for 1 h at room temperature, and the cell nuclei were counterstained with ProLong^TM^ Gold antifade reagent whit DAPI (Invitrogen). Photomicrographs were taken using an inverted fluorescence microscope (Nikon Eclipse Ts2R) coupled to a digital camera (Nikon DS-Ri2) and the images were computer-analyzed with NIS-Elements Br image software (Nikon). Fluorescence intensity measurement per MHC channel was used to assess the fluorescence of samples labeled with Alexa Green or Alexa Red. Prior to analysis, MHC mode fluorescence thresholds were set and applied to all images. The values obtained were normalized with respect to the number of cells per field. At least three replicates of each protein were performed.

### 4.7. Immunoblot for α-SMA, MMP1, MMP9, ERK, P-ERK, NF-κB and p-NF-κB

Cultures were RF- or sham-exposed for 12 or 24 h. At the end of electric treatment, the cells were lysed for protein extraction. The immunoblot procedure has been described in detail elsewhere [21,48]. Briefly, the protein samples (100 μg protein aliquots) were separated in 10% sodium dodecyl sulphate-polyacrylamide gel and electrophoretically transferred to nitrocellulose membrane (Amersham, Buckinghamshire, UK). Blots were incubated at 4 °C overnight in mouse monoclonal anti- α-smooth muscle actin antibody α-SMA (1:400; cat. no. A 2547; Sigma Aldrich), rabbit monoclonal anti-MMP-9 antibody (1:1000, cat. no. ab76003; Abcam, Cambridge, UK), rabbit monoclonal anti-MMP1 antibody (1:1000; cat. no. ab134184; Abcam), rabbit monoclonal anti-p44/42 MAPK (ERK1/2) (1:1000; cat.no. 4695; Cell Signaling, Danvers, MA, USA), rabbit polyclonal anti Phospho-ERK1/ERK2), mouse monoclonal anti-NF-ΚB p65 (f-6) (1:1000; cat.no.sc-800; Santa Cruz, CA, USA), and rabbit monoclonal anti-phospho-NF-κB p65 (Ser536) (93H1) (1:1000; cat.no. 3033; Cell Signaling). Rabbit polyclonal anti-GAPDH antibody (1:500, cat. No. sc-25778; Santa Cruz Biotechnology) was used as loading controls. The membranes were incubated for one hour at room temperature with anti-mouse IgG horseradish peroxidase conjugated antibody (1:10,000 NA931; GE Heathcare, Hatfield, UK) and with anti-rabbit IgG horseradish peroxidase conjugated antibody (1:5000 NA934; GE Heathcare). Then, the membranes were scanned with a Bio-Rad imaging system (Hercules, CA, USA). The obtained bands were densitometry evaluated (PDI Quantity One 4.5.2 software, BioRad). At least three experimental replicates were conducted per protein. All values were normalized over the loading control.

### 4.8. Statistical Analysis

All procedures and analyses were conducted in blind conditions for treatment. At least three independent replicates were conducted per experiment or exposure interval. Results were expressed as means ± standard deviation (SD) or standard error of the mean (SEM). Unpaired Student’s *t*-test was applied using GraphPad Prism 6.01 software (GraphPad Software, San Diego, CA, USA). Differences *p* < 0.05 were considered significant statistically.

## 5. Conclusions

This study reveals that CRET therapy could act on cutaneous fibrosis, reducing the fibrotic extracellular matrix, intervening in the control of the expression of the proteins involved in its degradation, and reducing the activation of pro-inflammatory transcription factors such as NFkB. Thus, this study provides some of the biological bases of the effects of CRET. However, although the results would suggest the utility of this therapy for the treatment of cutaneous fibrosis, a deeper understanding of the cellular and molecular mechanisms of response to CRET is necessary. On the other hand, this study was carried out in vitro, which is a limitation for direct extrapolation to patients. Thus, it is essential to carry out studies at the organism level and preferably in humans through clinical trials, to determine the real usefulness of CRET therapy in this pathology.

## Data Availability

The data presented in this study are available on request from the corresponding author. The data are not publicly available due to privacy restrictions.

## Supplementary Materials

The following supporting information can be downloaded at: https://www.mdpi.com/article/10.3390/ijms241310986/s1.
