# Supplementary material for: Anti-Fibrotic Effects of RF Electric Currents
Source: Int J Mol Sci. 2023 Jul 1;24(13):10986. doi: 10.3390/ijms241310986 (PMC10341950; doi:10.3390/ijms241310986)
Supplement: Supplementary file 1 [file ijms-24-10986-s001.zip › ijms-2418291-supplementary.pdf]

A

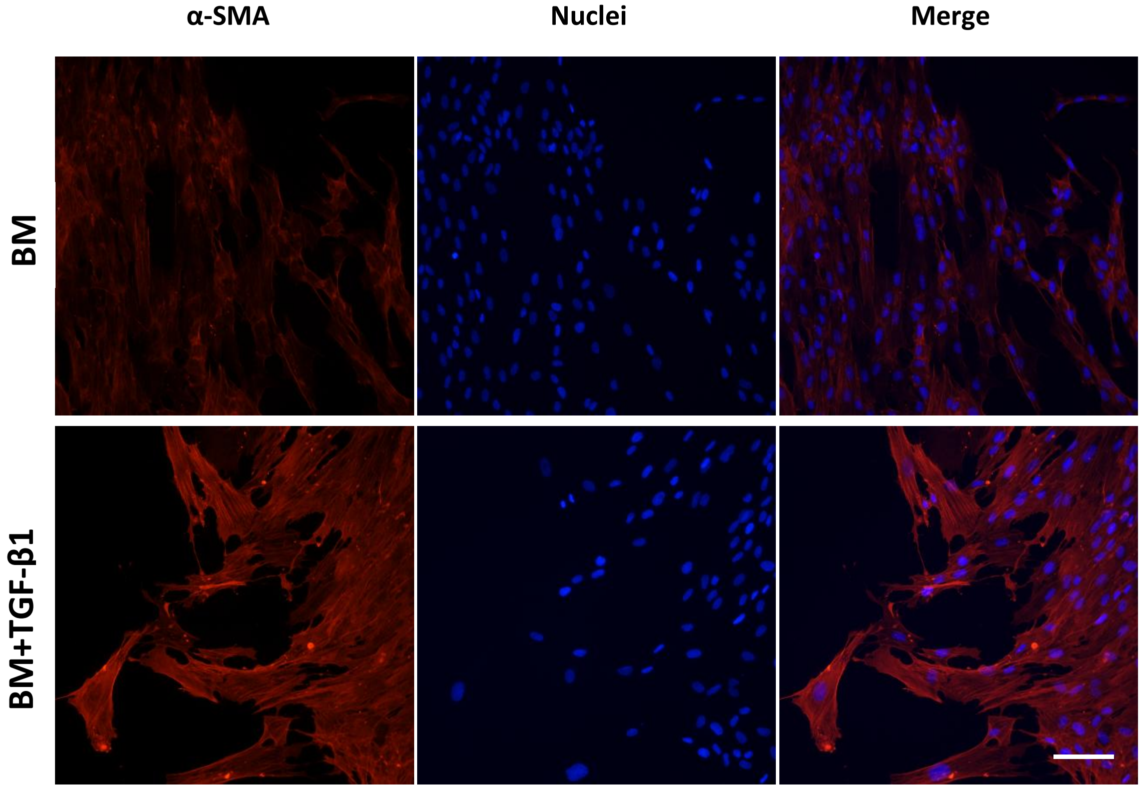

B

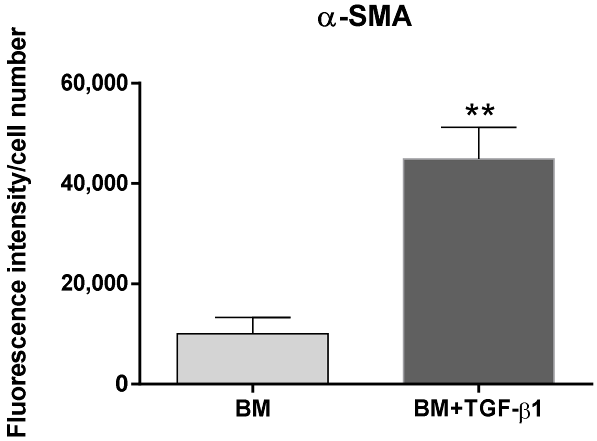

Supplementary Figure S1. Control of TGF-β1-induced myofibroblast differentiation. Fibroblasts cultured for 12 days with medium supplemented with TGF-β1 (2 ng/ml) (BM+TGF-β1) or maintained under the same experimental conditions with basal medium (BM). Scale bar: 20 μm. A. α-SMA immunofluorescence. 14 images evaluated per experimental group. B. Mean ± SEM of fluorescence/number of cells counted in the acquired images. **: $0.001 \leq p < 0.01$. T-Student test.
